# Evaluation of Spiral Ganglion Lesions by Electrophysiological Measures

**DOI:** 10.3390/brainsci16020140

**Published:** 2026-01-28

**Authors:** Max Meuser, Susanne Schwitzer, Parisa Sadat, Horst Hessel, Rainer Seidl, Philipp Mittmann, Dietmar Basta

**Affiliations:** 1 Department of Otolaryngology at UKB, Charité Medical School, University of Berlin, 12683 Berlin, Germany; 2 Cochlear Deutschland GmbH & Co. KG, Mailänder Str. 4a, 30539 Hanover, Germany

**Keywords:** cochlear implant, neural health, electrically evoked compound action potential, efficiency index

## Abstract

**Background:** Through the direct electrical stimulation of spiral ganglion neurons (SGNs) of the hearing nerve, cochlear implants overcome functionally impaired or missing hair cells in patients with profound to severe hearing loss. In routine clinical fitting, regions with severe local SGN degeneration (modiolar “dead regions”) cannot be identified. As a result, the electrical fields of neighboring electrodes are broadened, which can lead to increased channel interaction and, consequently, poorer speech understanding and hearing. The objective of this study was to ascertain whether neural health status can be evaluated by using cochlear implants’ inbuilt measures. **Methods:** Electrode impedance (MP1-, MP2-, MP1/MP2-, common ground mode), transimpedance matrix (TIM) and electrically evoked compound action potential (eCAP) measurements were performed before and after laser-induced induction of lesions on the modiolus of the guinea pig. Laser treatment-related shifts in impedance, TIM, and eCAP characteristics (threshold, amplitude, and a modified version of the failure index, referred to as the efficiency index (EI)) were correlated with the histologically assessed damage in three predefined areas of the basal modiolus within the electrode region. **Results:** Modiolar damage resulted in a significant reduction in the electrode impedance in MP2- and MP1/2-mode, the eCAP amplitude, and the EI. In contrast, TIM values and eCAP thresholds were significantly elevated. MP1, MP1/MP2 electrode impedance, TIM, and the eCAP thresholds were not correlated with the extent of modiolar damage. The shifts in eCAP amplitudes and the EI were significantly correlated with the damage at all regions of the basal modiolus. **Conclusions:** The eCAP amplitude and the EI are both capable of objectively evaluating the neural health status of the cochlea. Thus, a modiolar dead region could be expected from a local drop in eCAP amplitude values or the modified EI within the electrode array.

## 1. Introduction

For patients with profound to severe hearing loss, the use of a cochlear implant (CI) is the main treatment option. The CI circumvents the functionally impaired hair cells and directly stimulates the spiral ganglion neurons (SGNs) of the cochlear nerve. The functional efficacy of cochlear implants is thought to be closely related to cochlear neural health, which is the condition of the auditory nerve targeted by the device. A commonly used anatomical indicator for assessing auditory nerve integrity in research is the number or density of spiral ganglion neurons. Although the correlation between the number of surviving SGNs and CI outcomes is still under debate, studies suggest that the health of SGNs positively impacts speech understanding in implanted patients [1,2,3,4]. The survival rate of these neurons shows an enormous variation from about 2% to 72% in CI patients [5,6]. Besides this wide range in the total number of SGNs, heterogeneity in SGN degeneration or distribution along the length of the cochlea has been described in animals [7] and humans [8,9]. This phenomenon includes local SGN degeneration equivalent to audiologically known hair cell “dead regions” [10], where the function of SGNs is so severely compromised that a tone of a certain frequency results in off-place listening when presented at sufficiently high amplitude [11].

The current state of the CI processor’s fitting process does not encompass sufficient data pertaining to neural health, nor does it provide any indication of the correspondence between the position of a specific electrode contact and the location of a modiolar dead region (mDR). Thus, it can be anticipated that the increased activation of an array contact that is placed at an mDR is unavoidable in the CI fitting process. The resulting spread of excitation to healthy, unimpaired regions is consequently expected to culminate in off-place listening in mDR-harboring CI patients [12,13]. In line with this are results from CI patients showing that current focusing of channels that are suspected to have a high degree of channel interaction, or the deactivation of electrode contacts with broad nerve excitation, leads to better speech understanding [14,15]. Therefore, an urgent need for an electrophysiological measurement protocol to assess neural health and to identify mDRs in CI patients should be recognized. This method should be objective in order to be fast and reliable, and should provide appropriate care for young children or non-compliant patients.

Impedance and transimpedance matrix (TIM) are considered routine clinical measurements, providing information about the electrode position, changes in electrical properties of electrodes due to tissue alterations, and the tissue-electrode interface [16,17,18]. While these measurements permit conclusions to be drawn about the general status of intracochlear tissue (including the status of SGNs), the specificity of these non-neural measurements to assess the patient’s neural health is questionable. The measurement of electrically evoked compound action potentials (eCAPs), on the other hand, has been shown to be an objective method to detect the synchronized response specifically generated by electrically activated auditory nerve fibers. This method requires minimal patient cooperation and is not affected by the patient’s mental state [19]. In the past years, there has been increasing interest in employing eCAPs to assess neural health in CI patients. As the eCAP threshold is determined primarily by the smallest subgroup of the most synchronized and functional auditory nerve fibers capable of generating a measurable response, it does not necessarily reflect the condition of the entire neural population. Consequently, several studies in guinea pigs have shown that even substantial neural degeneration does not translate into marked changes in eCAP thresholds at two or six weeks after the onset of deafness [20]. This is consistent with findings by Pfingst et al. (2011) [21], who likewise observed no significant relationship between varying SGN densities and the measured eCAP threshold in guinea pigs.

Therefore, recent research has focused on other eCAP-based measurement parameters that could more reliably detect such correlations. In this regard, the slope of the eCAP amplitude—being directly influenced by the total number of responsive auditory nerve fibers—has repeatedly been shown to correlate with different degrees of neural survival [4,21]. However, it has been demonstrated that SGN density accounts for only 50% of the variance in the slope of the eCAP input/output function [22]. Furthermore, studies on human CI users yielded conflicting results when investigating the relationship between the slope of the eCAP input/output function and speech perception scores [23,24,25,26].

Artificial manipulation of neural health by induction of lesions in the modiolus is well described in cats [27,28,29]. Tone-evoked CAP audiograms before and after the lesion revealed elevated thresholds after the induction of a lesion. Furthermore, CAP audiograms revealed that the threshold shifts and the range of affected frequencies increase in proportion to the size of the lesion [29]. More recently, using a microelectrode, Konerding et al. (2022) [30] induced mechanical micro-lesions to the modiolus of guinea pigs to achieve targeted somatic and/or peripheral dendritic ablation of SGNs. The evaluation of eCAP measurements performed before and directly after the application of the lesion showed no significant changes in thresholds, maximum amplitude, or the slope of the growth function of the early N1–P1 component across all lesion types. However, the analysis of late eCAP components (N2–P2 ≈ 1 ms after stimulus onset), as described in rats [31] and guinea pigs [32,33], showed significantly elevated thresholds in the basal turn (near the lesion), but not apical to the lesion, whereas the amplitude and the slope of the growth function were reduced [30]. In contrast, a study of chronic peripheral and/or somatic lesions (eCAP measurement before and 8–12 days after the induction of a lesion) showed a significant increase in eCAP thresholds, as well as reduced amplitudes and slopes of the growth function of the N1–P1 component across all lesion types [34]. Recently, Konerding et al. (2025) [34] suggested the failure index (FI) as the input/output ratio at the maximal output to assess neural health of the cochlea. In guinea pigs, it was shown that the FI: (1) was significantly increased in the presence of SGN ablation; (2) was able to identify contacts closest to the lesion in approximately 80% of cases; and (3) was correlated with the size of the lesion. Furthermore, two recent clinical preprint studies provide evidence that the FI could be a robust estimate of neural health and may act as an early indicator of speech performance abilities in CI listeners, potentially guiding speech-processor fitting to optimize CI outcomes [35,36].

The present study investigates possible correlations of varying neural health statuses with the characteristics of cochlear implants’ in-built measurement methods. In contrast to human experiments, animal experiments enable a direct and acute comparison of conditions, as well as precise quantification of the lesion using histological methods. To this end, laser-induced degeneration of SGNs was generated at the modiolus of the guinea pig. Using histological analyses, the laser-induced damage was classified in the basal turn and correlated with electrode impedances and eCAP characteristics (eCAP threshold, amplitude of the growth function, and a modified version of the failure index, referred to as the efficiency index (EI)).

## 2. Methods

### 2.1. Animals

The experiments were carried out in seven normally hearing adult (17–24 weeks) guinea pigs (Dunkin Hartley, *Cavia porcellus*) of both sexes. The study was approved by the Governmental Commission for Animal Experiments (LaGeSo Berlin, Germany, approval No. G009023). The study was conducted in accordance with the EU Directive 2010/63/EU on the protection of animals used for scientific purposes. Every effort was made to minimize pain or discomfort.

All surgeries and measurements were performed under general anesthesia (ketamine (6 mg/100 g) and xylazine (0.6 mg/100 g), i.m.). The body temperature was maintained at 37 °C by a heating mat placed under the animal.

### 2.2. Surgical Procedure

**Figure 1:**
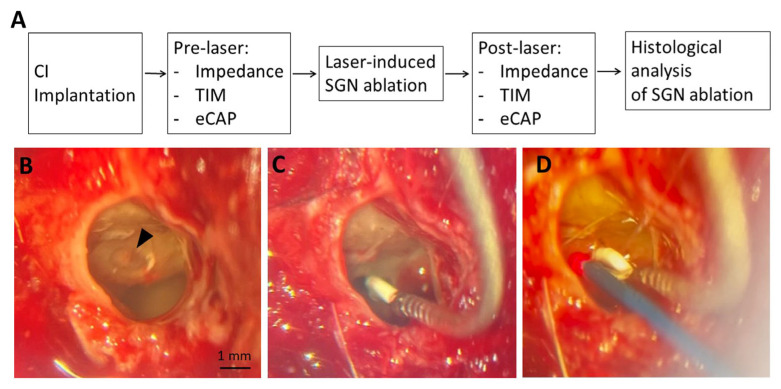
Experimental procedure (**A**). A basal cochleostomy (arrowhead) was drilled to fit the CI electrode and the laser fiber (**B**). The CI electrode was inserted and pre-laser measurements were performed (**C**). The laser was inserted while the CI electrode remained in the cochlea. The laser was pointed towards the modiolus and laser treatment was applied to produce dead regions (**D**). eCAP = electrically evoked compound action potential; SGN = spiral ganglion neurons; TIM = transimpedance matrix.

After opening the bulla, an oval cochleostomy was drilled in the basal turn of the cochlea. Via this window, a customized guinea pig scala tympani electrode array with eight electrode contacts (Animal Array HL8, Cochlear, Sydney, Australia) was inserted (Figure 1B,C). The electrode array was connected to a CL24RE Implant Emulator (Cochlear, Sydney, Australia). The cochleostomy was sealed with muscle tissue, and the extracochlear reference electrode MP1 (multi-polar 1) was placed at a ventral position, whereas the MP2 (multi-polar 2) electrode was placed in a rostral position. Impedance and eCAP measurements were performed, and the muscle tissue was removed subsequently. A surgical laser fiber (fiber diameter: 400 µm, diode laser 980 ± 30 nm; CW 25 W, Biolitec, Jena, Germany) was carefully inserted into the cochleostomy without moving the electrode array (Figure 1D). To induce SGN degeneration, the laser fiber was aligned to the modiolus and laser treatment was performed for 1 s at 5 W (resulting in a power of approx. 5 J). The laser fiber was carefully removed, and the cochleostomy was covered again with muscle tissue. Finally, impedance and eCAP measurements were repeated.

### 2.3. Electrophysiological Measurements

eCAP, impedance, and transimpedance matrix measurements (TIM) were performed just before and directly after the laser treatment, using the Custom Sound EP 6.0 software with the masker–probe algorithm. Impedance measurements were performed at the MP1 (measuring the resistance between the corresponding array contact and the MP1 electrode located at a ventral position), MP2 (measuring the impedance between the corresponding array contact and the MP2 electrode located at a rostral position), the combined MP1/MP2, and common ground (CG) (measuring the impedance between one active intracochlear array contact with all remaining contacts acting as a shared return) modes. Transimpedance matrix measurements were performed at all eight electrodes in the “MP1 Stim − MP2 Rec” mode (4-point mode) with a pulse width of 25 µs at 180 current level (CL). The measurement point was T6—the end of the negative phase.

ECAPs were measured with a pulse width of 25 µs applied for both the masker and the probe, using a masker–probe interval of 400 µs. Fifty sweeps were recorded with a gain of 40 dB and a delay of 68 µs. Stimulus current was increased by 5-CL steps from 180 to 220 CL. The threshold level was determined as the level of the last visually detected neural response consistently by the same expert.

The conversion from current level in µA was calculated using the logarithmic CL-to-current mapping.

### 2.4. Histology

Immediately following the final eCAP measurement, tissue fixation was performed by perfusion with a 4% paraformaldehyde (PFA) solution (pH 7.4, Sigma, Taufkirchen, Germany) via the left heart chamber. The cochleae were carefully extracted and incubated in 4% PFA overnight at 4 °C. The samples were stored in 0.1% PFA at 4 °C until further analysis.

For decalcification, the cochleae were immersed in 0.4 M EDTA for 14 days, with the solution being replaced every other day. Following decalcification, the cochleae were embedded in paraffin, and 6 μm frontal sections were prepared using a sliding microtome (Leica SM2000R, Leica Biosystems, Nußloch, Germany). The sections were stained following a modified hematoxylin-eosin (H&E) protocol. The slides were treated twice with Roti-Histol (Carl Roth, Karlsruhe, Germany) for 5 min each to remove the paraffin. The rehydration process was conducted via a graduated ethanol series (90%, 70%) and distilled water, each for 5 min. The nuclear staining procedure involved the use of Mayer’s hematoxylin for 5 min, followed by a 10-min differentiation process under running tap water. The cytoplasmic staining was executed using 0.1% eosin for 50 s, followed by a brief rinsing in distilled water and differentiation in 70% ethanol. Dehydration was achieved using an ascending ethanol series (90% ethanol, isopropanol), followed by clearing in Roti-Histol. Finally, the slides were mounted for further analysis using the Roti-Histokit (Carl Roth, Karlsruhe, Germany).

### 2.5. Classification and Weighting of Laser-Induced SGN Ablation of the Modiolus

In order to evaluate the laser-induced intracochlear damage to the modiolus, the basal part of the modiolus was separated into three areas: basal 1 (most basal area), basal 2 (second most basal area), and basal 3 (most apical area).

The damage in these areas was initially classified in each sample according to the severity of the observed damage: class 1: loss of <50% SGNs, class 2: loss of ≥50% of the SGNs in the basal turn, and class 3: complete loss of SGNs or complete ablation of the basal portion of the auditory nerve. The length of the damage in each class was then calculated by multiplying the number of sections on which the respective damage was detected by the section thickness (6 µm). The SGN ablation was weighted by multiplying the length of the damaged area by the corresponding damage class (Table 1). Thus, each modiolus area of each sample was assigned its own damage score, determined by the classification-based weighting of the damage.

### 2.6. Determination of the Regions of the Basal Modiolus and Their Assignment to Array Contacts

**Figure 2:**
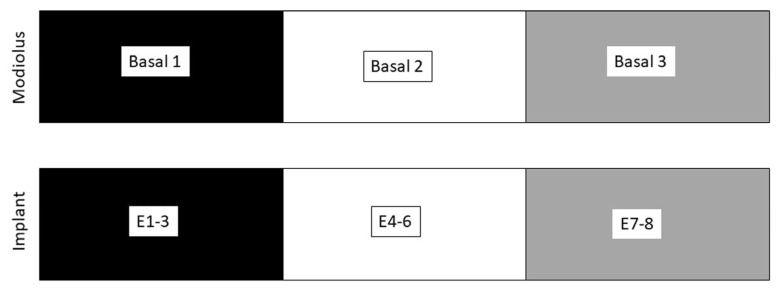
Schematic representation of the modiolus and the implant indicating the spatial assignment of the array contacts (E1–E8) to the corresponding areas of the basal modiolus (basal 1–3).

Based on anatomical landmarks (cochleostomy, round window membrane), the basal modiolus was divided into a most basal region (basal 1), a second most basal region (basal 2), and a more apical region (basal 3). In this regard, the most basal contacts of the array (E1–E3) were assigned to basal 1. The intermediate contacts of the array (E4–E6) were assigned to the basal 2 region, and the most apical contacts (E7–E8) were assigned to the most apical region of the basal modiolus, basal 3 (Figure 2).

### 2.7. Introduction of the Efficiency Index as a Modified Form of the Failure Index

The failure index (FI) is a marker of the efficiency of neural activation that was initially defined by Konerding et al. (2025) [34] as the input/output ratio at the maximal output:

(1) $$FI=\frac{{current}_{Max}}{\max amplitude}$$

The application of this equation results in an FI with a value of zero being assumed to be the most efficient FI. However, this does not allow for the integration of contacts that are characterized by a total absence of the neural response. In order to include array contacts without an eCAP response (amplitude = 0) in the analysis, the equation was modified and the FI was renamed as follows:

(2) $$Efficiency\;index\;(EI)=\frac{\max amplitude}{{current}_{Max}}$$

This modification sets zero as the threshold value representing the least efficient neural activation and allows the inclusion of electrode contacts for which no neural response could be measured.

### 2.8. Statistical Analysis

SPSS software (IBM SPSS Statistics version 25, IBM Corp., Armonk, NY, USA) was used for all statistical analyses. Data distribution was tested with the Shapiro–Wilk test.

Fourteen samples were included in all statistical analyses at the basal 1 and basal 2 areas of the modiolus. All analyses at the most apical area, basal 3, were performed with 10 samples, since four samples with imperfect insertion needed to be excluded, as it cannot be expected that array contacts reached the basal 3 area of the modiolus in these samples. All analyses were performed with the mean values of the array contacts E1–E3, E4–E6, and E7–E8. Potential outliers were identified using a two-sided Grubbs’ test (α = 0.05). Values exceeding the critical Grubbs’ statistic were classified as outliers and excluded prior to statistical comparison.

The comparison of pre- and post-laser treatment means of the impedance factors, TIM, the eCAP thresholds, the N1–P1 amplitudes, and the EI was performed using either the Wilcoxon signed-rank test or a paired *t*-test.

For the evaluation of possible correlations between the damage score and the shift in impedance factors, TIM, and eCAP characteristics (eCAP threshold, amplitude of the growth function, and EI), shifts were calculated as the difference between the pre- and post-laser mean values. The most basal array contacts E1–E3 were assigned to the most basal area in the basal turn of the modiolus (basal 1). The contacts E4–E6, located in the center of the array, were assigned to the second most basal area of the basal turn of the modiolus (basal 2), and the contacts E7–E8, located at the apex of the array, were assigned to the most apical area in the basal turn of the modiolus (basal 3) (Figure 2). The correlation analyses were performed by either a Pearson’s correlation analysis or Spearman’s rho, depending on data distribution. The significance level for all statistical tests was set at *p* ≤ 0.05. Multiple-comparison correction was performed using the Bonferroni procedure by adjusting the *p*-values to control the family-wise error rate. Linear regression lines were displayed only when the correlation was significant (black solid line) or when the data visually supported a linear relationship (grey, dashed line). In these cases, ordinary least squares regression was used and 95% confidence bands were calculated based on the standard error of the estimate and the t-distribution (n − 2 degrees of freedom).

## 3. Results

### 3.1. Laser-Induced Damage at the Basal Part of the Modiolus

**Figure 3:**
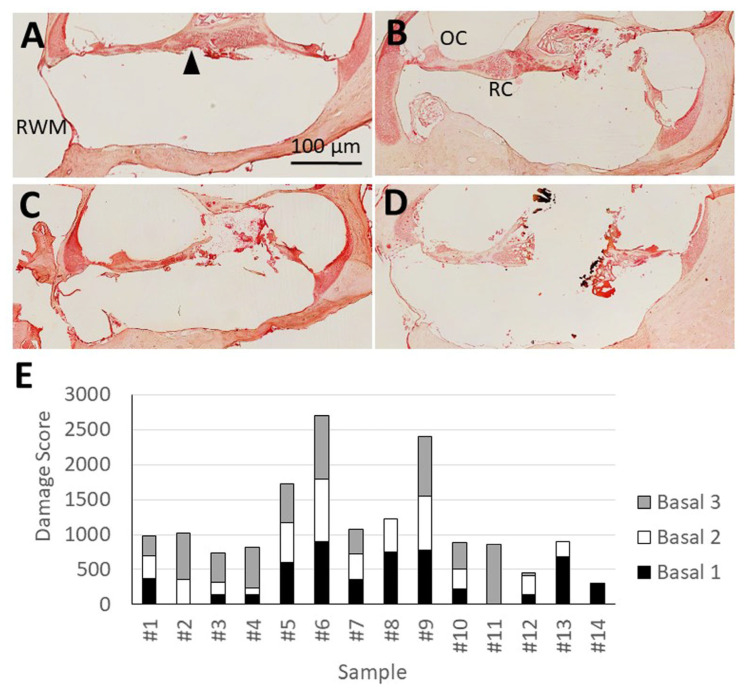
Laser-induced intracochlear damage to the basal modiolus. Example of mild damage with a loss of SGNs of less than 50% in the basal turn (arrowhead) (**A**), a loss of SGNs of ≥50% (**B**); a complete loss of SGNs in the basal turn (**C**) and a complete loss of the basal portion of the cochlear nerve (**D**). (**E**) Damage scores at the different portions of the modiolus in the basal turn (Basal 1–3) for each sample. OC = organ of Corti; RC = Rosenthal’s canal; RWM = round window membrane; SGN = spiral ganglion neuron.

Histological analyses demonstrated that the application of a laser beam resulted in varying degrees of damage to the basal part of the modiolus. Three distinguishable classes of damage were identified: class 1 showed slight damage to the modiolus (loss of less than 50% SGNs), class 2 exhibited strong damage (loss of 50–99% of SGNs), and class 3 revealed a complete loss of SGNs or a complete loss of the basal portion of the auditory nerve (Figure 3). The evaluation of the extent of damage and the subsequent weighting of these lengths by the classes of SGN ablation revealed a set of distinct damage patterns among all examined regions of the basal modiolus of all samples analyzed. The resulting damage scores of the samples ranged from 0 to 900 at basal 1 (SD ± 292), basal 2 (SD ± 255), and basal 3 (SD ± 319) (Figure 3).

### 3.2. Influence of Laser-Induced SGN Ablation on Impedance Characteristics and TIM

The comparison of impedance characteristics before and after the laser-induced SGN ablation at the three different areas of the basal modiolus revealed values between 3.27 kΩ and 8.82 kΩ (SD ± 1.5 kΩ) in the MP1 mode at E1–E3 before, and 2.58 kΩ to 7.25 kΩ (SD ± 1.4 kΩ) after the laser treatment. At E4–E6, 4.33 kΩ to 7.59 kΩ (SD ± 0.95 kΩ) were measured in the MP1 mode before, and 4.02 kΩ to 7.35 kΩ (SD ± 1 kΩ) after the laser treatment. At E7–E8, 3.77 kΩ to 13.49 kΩ (SD ± 2.31 kΩ) were obtained in MP1 before, and 3.73 kΩ to 9.41 kΩ (SD ± 1.56 kΩ) after the laser treatment. None of these differences were significant.

In the MP2 mode, impedance between 2.65 kΩ and 8.51 kΩ (SD ± 1.59 kΩ) was measured at E1–E3 before, and 2.41 kΩ to 7.2 kΩ (SD ± 1.37 kΩ) after the laser treatment. This difference was not significant. At E4–E6, a significant difference was found in the impedance with values between 4.68 kΩ and 9.96 kΩ (SD ± 1.42 kΩ) in the MP2 mode before, and 4.09 kΩ to 7.43 kΩ (SD ± 1.09 kΩ) after the laser treatment (*p* = 0.049). At E7–E8, values between 4.38 kΩ and 13.19 kΩ (SD ± 2.28 kΩ) before, and 4.46 kΩ to 9.57 kΩ (SD ± 1.66 kΩ) after the laser-induced destruction indicated a significant reduction in impedance in this mode at this area of the basal modiolus (*p* = 0.022).

In the MP1/MP2 mode, impedance between 2.62 kΩ and 8.1 kΩ (SD ± 1.15 kΩ) was observed before, and 2.33 kΩ to 6.8 kΩ (SD ± 1.27 kΩ) after the treatment at E1–E3. At E4–E6, values between 4.09 kΩ and 7.18 kΩ (SD ± 1.4 kΩ) were found before, and 3.79 kΩ to 6.87 kΩ (SD ± 0.97 kΩ) after the laser treatment. These differences were not significant. At E7–E8, a significant reduction in impedance was found, represented by impedance values between 3.53 kΩ and 12.77 kΩ (SD ± 2.27 kΩ) before the laser treatment, and 3.48 kΩ to 8.98 kΩ (SD ± 1.6 kΩ) after (*p* = 0.029).

The measurement of the common ground impedance (CG) revealed 1.42 kΩ to 7.43 kΩ (SD ± 1.71 kΩ) at E1–E3 before the laser treatment, and 2.0 kΩ to 4.96 kΩ (SD ± 1.26 kΩ) thereafter. At E4–E6, CG values between 2.86 kΩ and 5.58 kΩ (SD ± 1.26 kΩ) were found before, and 2.82 kΩ to 5.41 kΩ (SD ± 0.8 kΩ) after the laser-induced destruction at the modiolus. At E7–E8, the measured CG impedance was 2.67 kΩ to 11.39 kΩ (SD ± 2.67 kΩ) before, and 2.56 kΩ to 7.08 kΩ (SD ± 1.42 kΩ) after the laser treatment. None of these differences were significant.

**Figure 4:**
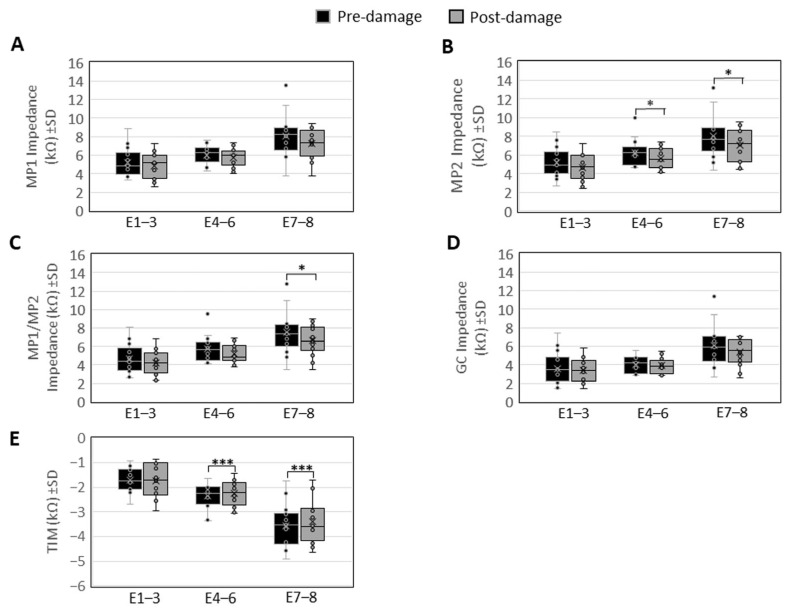
Comparison of impedance measurements before and after laser-induced damage to the modiolus. Shown are the means of the array contacts E1–3, E4–6 and E7–8 (±SD) for (**A**) the MP1, (**B**) the MP2, (**C**) the MP1/MP2, (**D**) the common ground (CG) component of the impedance, and (**E**) the transimpedance matrix (TIM). * = *p* ≤ 0.05; *** = *p* ≤ 0.001.

Transimpedance matrix measurements revealed values between −2.67 kΩ and −0.94 kΩ (SD ± 0.46 kΩ) at E1–E3 before, and −2.96 kΩ to −0.86 kΩ (SD ± 0.65 kΩ) after the treatment. At E4–E6, TIM measurements revealed −3.35 kΩ to −1.65 kΩ (SD ± 0.49 kΩ) before, and −3.06 kΩ to −1.44 kΩ (SD ± 0.51 kΩ) after the damage at the modiolus. At E7–E8, TIM values between −1.7 kΩ and −4.9 kΩ (SD ± 0.85 kΩ) were found before the laser treatment, and −4.64 kΩ to −1.7 kΩ (SD ± 0.83 kΩ) thereafter (Figure 4).

### 3.3. Influence of Laser-Induced Damage on eCAP Characteristics

**Figure 5:**
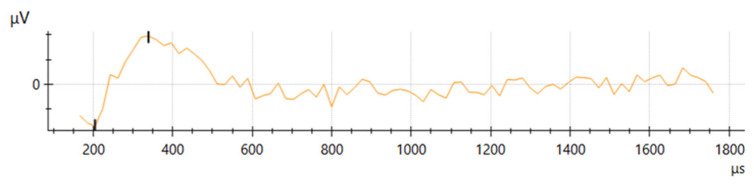
Representative electrically evoked compound action potential (eCAP) elicited by stimulation of spiral ganglion neurons at a single electrode contact within the cochlear implant array. The *y*-axis depicts the recorded neural response amplitude (µV), and the *x*-axis indicates time relative to stimulus onset (µs).

A representative example of the electrically evoked compound action potential recorded in this study is shown in Figure 5.

eCAP measurements at E1–E3 revealed a significant elevation of the thresholds, represented by values between 368.85 µA and 601.23 µA (SD ± 61.36 µA) before, and 407.19 µA to 930.1 µA (SD ± 184.61 µA) after the laser treatment (*p* = 0.002). At E4–E6, thresholds between 325.87 µA and 497.03 µA (SD ± 51.23 µA) were found before, and 337.91 µA to 930.1 µA (SD ± 141.25 µA) after the laser treatment. At E7–E8, thresholds between 145.69 µA and 459.48 µA (SD ± 100.98 µA) before, and 192.93 µA to 930.1 µA (SD ± 239.31 µA) were observed. None of the latter changes in the thresholds were significant.

The evaluation of the amplitude of the growth function revealed significantly reduced amplitudes at E1–E3, represented by values between 227.08 µV and 2620.87 µV (SD ± 731.13 µV) before the laser treatment, and 0 µV to 921.77 µV (SD ± 271.84 µV) thereafter (*p* = 0.019). At E4–E6, eCAP amplitudes were significantly reduced from 531.52 µV to 3758.38 µV (SD ± 937.53 µV) before the laser-induced damage at the modiolus, to 129.74 µV to 1899.88 µV (SD ± 540.96 µV) after the treatment (*p* = 0.009). eCAP amplitudes were reduced significantly also at E7–E8, represented by values ranging from 290.75 µV to 3332.56 µV (SD ± 1123.24 µV) before the laser treatment, to 178.84 µV to 850.72 µV (SD ± 202.32 µV) after the treatment (*p* = 0.026).

**Figure 6:**
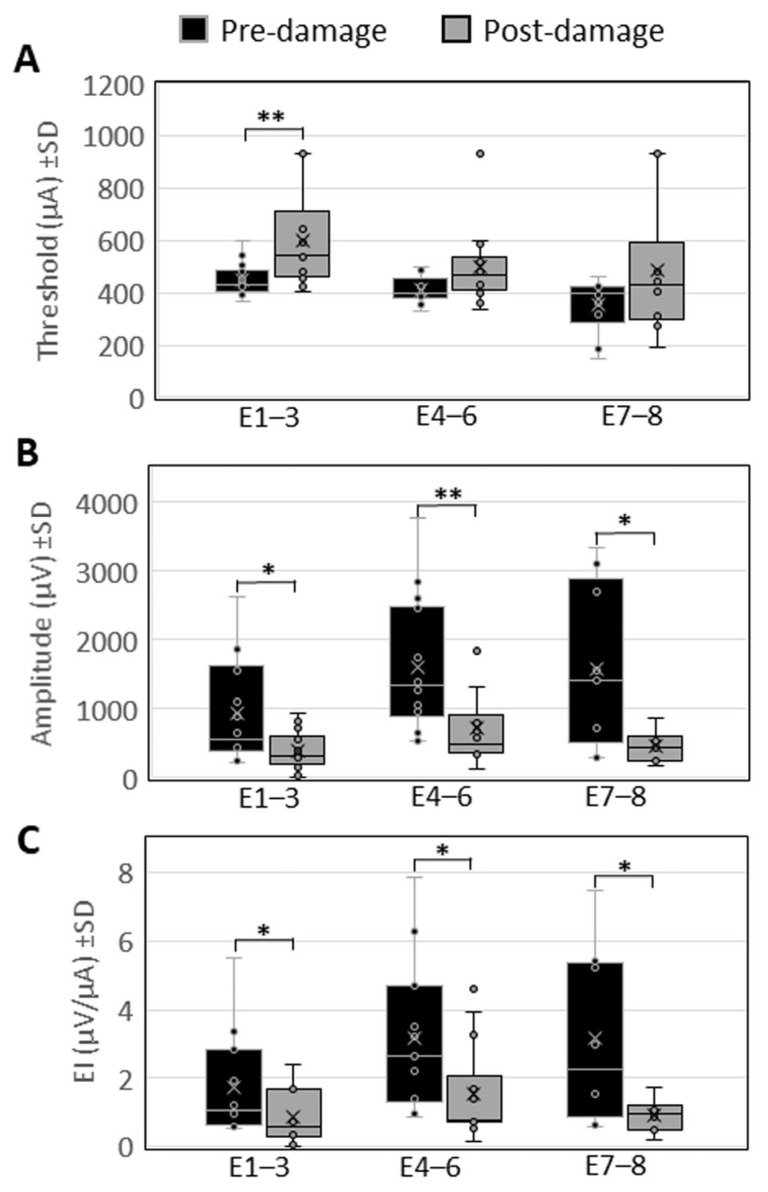
Comparison of the eCAP threshold (**A**), the amplitude (**B**) and the efficiency index (EI) (**C**) before and after laser-induced damage to the modiolus. Shown are the means of the array contacts E1–E3, E4–E6, and E7–E8 (± SD). * = *p* < 0.05; ** = *p* < 0.01.

The efficiency index (EI) was significantly reduced at E1–E3, with pre-laser treatment values between 0.51 µV/µA and 5.53 µV/µA (SD ± 1.4 µV/µA), and post-treatment values between 0 µV/µA and 2.38 µV/µA (SD ± 0.72 µV/µA) (*p* = 0.041). At E4–E6, a significantly reduced EI was observed, represented by values between 0.88 µV/µA and 7.88 µV/µA (SD ± 2.03 µV/µA) before, and 0.14 µV/µA to 4.62 µV/µA (SD ± 1.33 µV/µA) after the laser-induced damage at the modiolus (*p* = 0.019). At E7–E8, the EI was significantly reduced from pre-laser EI values between 0.57 µV/µA and 7.5 µV/µA (SD ± 2.41 µV/µA), to post-laser EI values between 0.2 µV/µA and 1.74 µV/µA (SD ± 0.46 µV/µA) (*p* = 0.046) (Figure 6).

### 3.4. Correlation Analysis of Impedance and TIM Shifts with the Extent of Modiolar Damage

**Figure 7:**
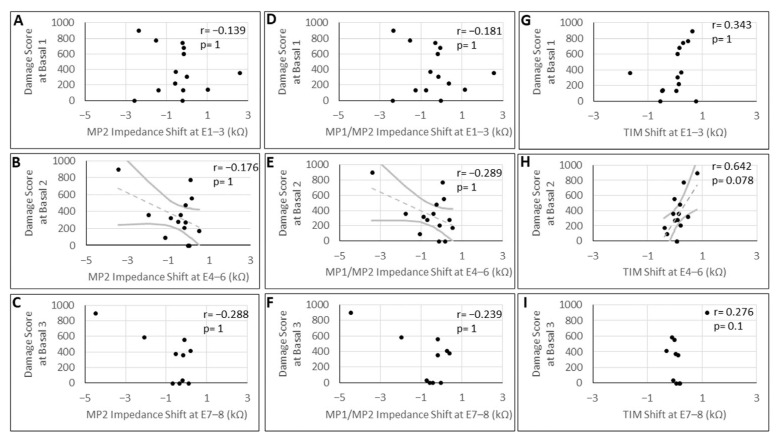
Correlation analysis between the mean shift in the impedance factors MP2 (**A**–**C**), MP1/MP2 (**D**–**F**), and the transimpedance matrix (TIM) (**G**–**I**) at the indicated array contacts (E1–E8) and the damage score at the basal 1–3 areas of the modiolus. Shaded areas denote 95% confidence bands. Gray dashed lines indicate linear regression fits that did not reach statistical significance. r = correlation coefficient; *p*-values were Bonferroni-adjusted for six comparisons.

Correlation analysis of the shifts in impedance factors and TIM, which underwent significant changes after laser-induced damage of the modiolus, and the damage score observed in the corresponding regions of the modiolus (basal 1–3) was performed. This analysis showed no significant correlation for the MP2 impedance shifts at E1–E3 (−2.85 kΩ to 2.58 kΩ (SD ± 1.35 kΩ)), at E4–E6 (−3.44 kΩ to 0.48 kΩ (SD ± 1.07 kΩ)), or at E7–E8 (−4.5 kΩ to 0.42 kΩ (SD ± 1.42 kΩ)). Furthermore, shifts in the MP1/MP2 impedance did not significantly correlate with the extent of damage at E1–E3 (−2.4 kΩ to 2.53 kΩ (SD ± 1.25 kΩ)), E4–E6 (−3.41 kΩ to 0.53 kΩ (SD ± 0.99 kΩ)), or E7–E8 (−4.47 kΩ to 0.36 kΩ (SD ± 1.37 kΩ)). In addition, the shifts in TIM measurements showed no such correlation at E1–E3 (−1.66 kΩ to 0.76 kΩ (SD ± 0.59 kΩ)), E4–E6 (−0.39 kΩ to 0.8 kΩ (SD ± 0.28 kΩ)), or E7–E8 (−0.31 kΩ to 1.17 kΩ (SD ± 0.37 kΩ)) to the damage at the three areas of the modiolus (Figure 7).

### 3.5. Correlation Analysis of eCAP Threshold Shifts with Damage Scores

**Figure 8:**
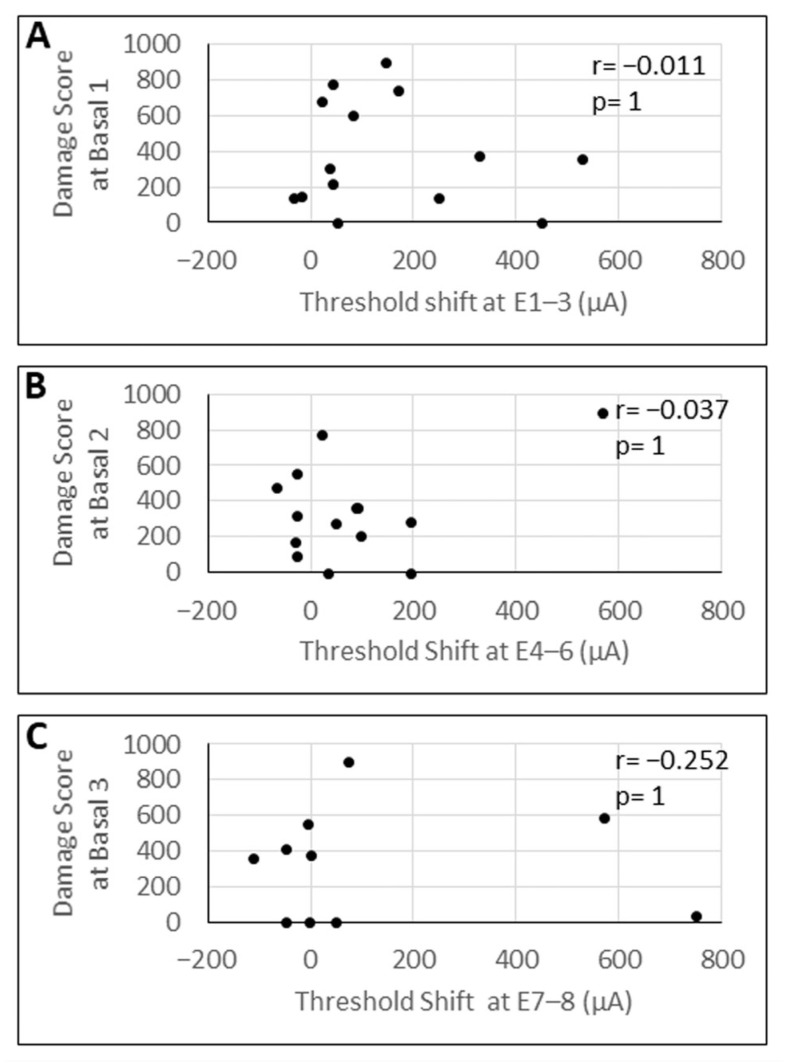
Correlation analysis between the mean shift in the eCAP threshold at the indicated array contacts (E1–E8) and the corresponding damage score at the basal 1 (**A**), basal 2 (**B**), and basal 3 (**C**) area of the modiolus. r = correlation coefficient; *p*-values were Bonferroni-adjusted for six comparisons.

The shift in eCAP thresholds after laser treatment ranged from −32.42 µA to 527.65 µA (SD ± 169.65 µA) at basal 1, from −66.34 µA to 565.92 µA (SD ± 155.12 µA) at basal 2, and from −113.43 µA to 747.54 µA (SD ± 275.85 µA) at basal 3. The correlation analysis between the shift in eCAP thresholds before and after the laser treatment and the damage score exhibited no significant correlation between these two factors at any of the investigated areas of the basal modiolus (Figure 8).

### 3.6. Correlation Between N1–P1 Amplitude Shifts and Damage Scores

**Figure 9:**
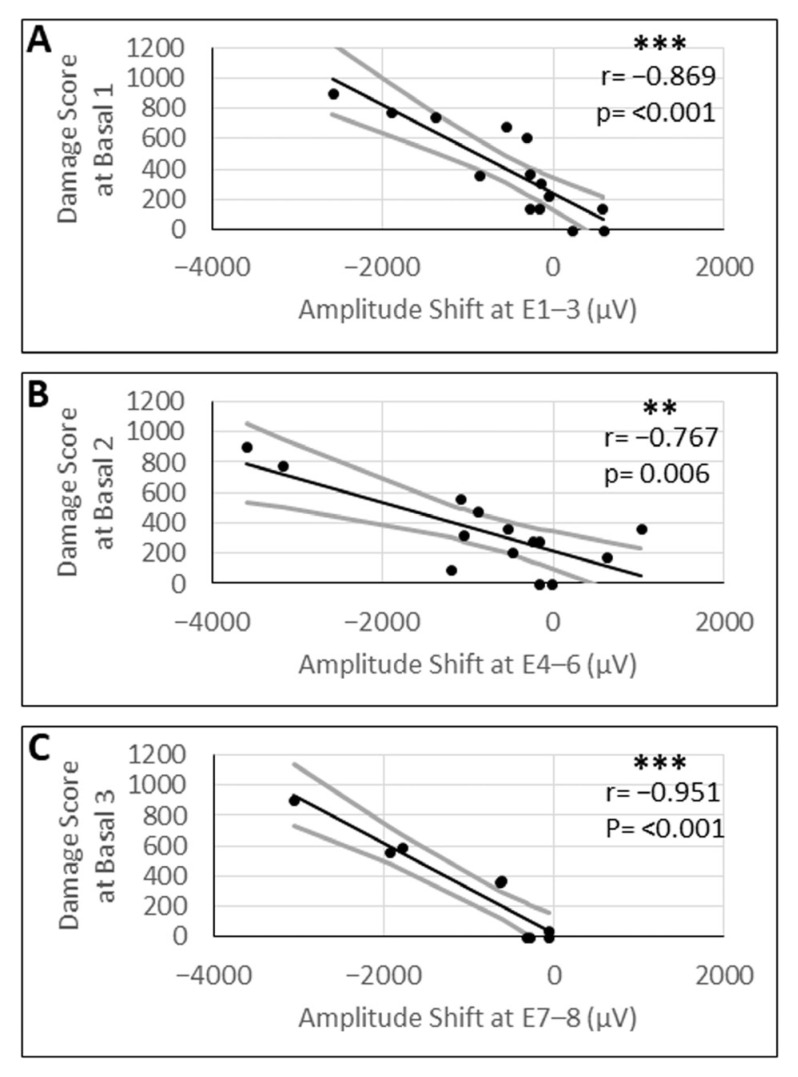
Correlation analysis between the mean shift in the eCAP amplitude at the indicated array contacts (E1–E8) and the corresponding damage score at the basal 1 (**A**), basal 2 (**B**), and basal 3 (**C**) region of the modiolus. Shaded areas denote 95% confidence bands. Black solid lines indicate statistically significant linear regressions. r = correlation coefficient; *p*-values were Bonferroni-adjusted for six comparisons. ** = *p* ≤ 0.01; *** = *p* ≤ 0.001.

The shift in the eCAP N1–P1 amplitudes of the growth function ranged from −1898.1 µV to 587 µV (SD ± 875 µV) at basal 1, from −3602.6 µV to 1028.8 µV (SD ± 1227.7 µV) at basal 2, and from −3054 µV to −59.08 µV (SD ± 983.49 µV) at basal 3. Pearson’s correlation analysis between the mean shift in N1–P1 amplitudes before and after the laser treatment and the damage score exhibited a significant negative correlation between these two variables at basal 1 (r = −0.86; *p* < 0.001), basal 2 (r = −0.76; *p* = 0.004), and the basal 3 region (r = −0.95; *p* < 0.001) of the basal modiolus (Figure 9).

### 3.7. Correlation Between Efficiency Index Shifts and Damage Scores

**Figure 10:**
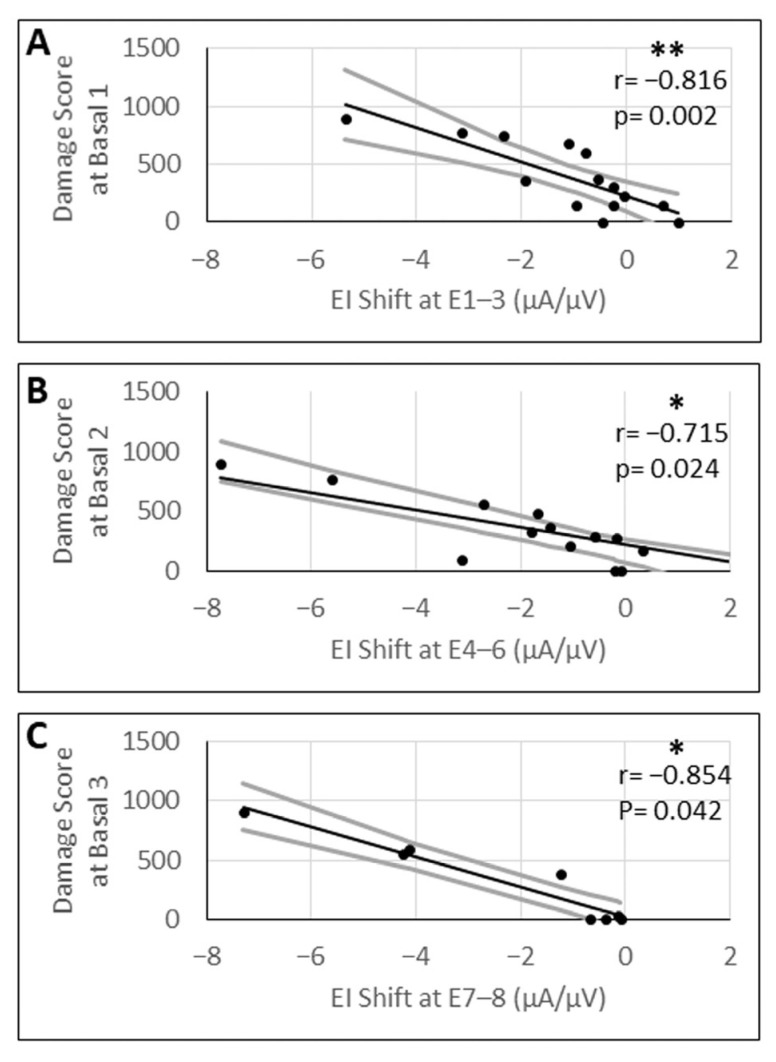
Correlation analysis between the mean shift in the efficiency index (EI) at the indicated array contacts (E1–E8) and the corresponding damage score at the basal 1 (**A**), basal 2 (**B**), and basal 3 (**C**) area of the modiolus. Shaded areas denote 95% confidence bands. Black solid lines indicate statistically significant linear regressions. r = correlation coefficient; *p*-values were Bonferroni-adjusted for six comparisons. * = *p* ≤ 0.05; ** = *p* ≤ 0.01.

The shift in the efficiency index after the laser treatment ranged from −5.35 µV/µA to 0.97 µV/µA (SD ± 1.59 µV/µA) at basal 1, from −7.73 µV/µA to 3.03 µV/µA (SD ± 2.53 µV/µA) at basal 2, and from −7.3 µV/µA to −0.08 µV/µA (SD ± 2.48 µV/µA) at basal 3. Pearson’s correlation analysis between the shift in the efficiency index before and after the laser treatment and the damage score exhibited a significant negative correlation between these two variables at basal 1 (r = −0.816; *p* = 0.002), basal 2 (r = −0.715; *p* = 0.024), and basal 3 (r = −0.854; *p* = 0.042) (Figure 10).

## 4. Discussion

### 4.1. Laser-Induced Introduction of SGN Degeneration

By applying a laser beam to the modiolus for a defined time and with a defined power, it was possible to generate divergent patterns of SGN degeneration ranging from a mild loss of SGNs to a total ablation of SGNs in the Rosenthal canal and/or the cochlear nerve (axonal fibers) in three areas of the basal turn. This relatively wide range can be explained by the fact that the exact orientation of the laser fiber in the basal turn of the cochlea and its exact position in relation to the modiolus was not apparent and therefore variable within a certain range. Consequently, a diverse pattern in terms of location and extent of SGN ablation can be expected and was even intended to induce divergent modiolar dead regions.

### 4.2. Electrode Impedance Is Reduced by the Laser Treatment but Does Not Correlate with the Extent of SGN Ablation at the Modiolus

A significant reduction in the MP2 and MP1/MP2 impedance observed in some areas of the basal modiolus indicates changes in the electrode–tissue interface (including the status of SGNs) caused by the laser treatment. Since the position of the CI array was not changed between the pre- and post-laser measurements, these changes should be caused by laser-induced tissue alterations, the composition of the cochlear fluids, and/or the temperature increase within the cochlea. In the context of radiofrequency catheter ablation, it is well documented that an increase in temperature at an electrode–tissue interface leads to tissue coagulation, necrosis, and a rise in ion mobility, resulting in a reduced impedance [37,38,39,40,41]. Since only the MP2 electrode showed significantly altered impedance, we conclude that the laser-induced tissue alterations only affected the current flow from the intracochlear contacts of the CI array to the rostrally positioned MP2 electrode. This could be related to the main target of the laser-induced tissue damage, which was the modiolar region. The region was reached by a laser beam in the rostral direction.

The significant elevation of TIM measurements further supports the idea that changed electrical properties are caused by general, but not neural-specific, tissue alterations, since none of the significant changes in impedance or TIM correlate with the extent of SGN ablation at the basal modiolus. We therefore conclude that impedance and TIM reflect the overall laser-induced tissue or fluid alterations, but are not sensitive measures for assessing the neural health of the cochlea.

### 4.3. Significantly Shifted eCAP Thresholds and Amplitudes After Laser-Induced Damage at the Modiolus

A significant increase in eCAP thresholds was found only in the most basal part of the modiolus. Furthermore, eCAP amplitudes were significantly reduced in all regions of the basal modiolus. This is in line with experiments in cats, showing elevated thresholds in tone-evoked CAP audiograms after the induction of a lesion [29]. Furthermore, guinea pig experiments showed a decrease in eCAP amplitudes 6 weeks after pharmacologically induced secondary loss of SGNs [20,33]. However, no significant changes in the eCAP thresholds were observed after secondary loss of SGNs in a model of furosemide-induced hearing loss [20]. Additionally, a study from Konerding et al. (2022) [30] showed no significant changes in the early N1–P1-generated threshold or the amplitude of the eCAP growth function after the induction of acute somatic and/or peripheral lesions. In this study, the analysis was performed across lesion types and therefore may have introduced a masking effect due to preserved peripheral dendrites in the somatic lesion group. Given that early eCAP components are primarily generated by peripheral SGN processes [42], one could hypothesize that a more selective dendritic lesion model might reveal higher sensitivity of the N1–P1 component to peripheral degeneration. Interestingly, a study of chronic peripheral and/or somatic lesions reflected the present finding of a significant increase in eCAP thresholds as well as reduced amplitudes of the N1–P1 component only if the data from all lesion types were pooled for the analysis [34]. That this holds true in the mentioned study, even for locally damaged regions, may be explained by the chronic nature of the lesion model, in which the eCAP recordings were performed (8–12 days after the insult). During this time window, secondary degenerative processes, most notably Wallerian degeneration, may have affected the peripheral SGN fibers despite the initial lesion being limited to the somatic region (for review see [43]). This would explain why early eCAP responses, which are typically considered insensitive to central or somatic injury, were nonetheless significantly altered in the chronic post-lesion condition. In this sense, the high sensitivity of the N1–P1 component of the eCAP measurements that we have observed suggests the consistent damage of peripheral dendrites in our laser-based SGN ablation model. This seems a reasonable assumption, as the anatomical position of the peripheral dendrites should lead to a particular exposure to the laser, applying a relatively intense power of approx. 5 J. As SGN damage consistently begins at the peripheral dendrites [44,45], our model appears to closely resemble the clinical manifestation of SGN loss.

### 4.4. The eCAP-Derived Efficiency Index Was Significantly Changed After Laser-Induced Damage to the Modiolus

The failure index suggested by Konerding et al. (2025) [34] is a measure of the efficiency of neural activation by an electrical impulse. An FI of zero defines the optimal effectiveness, whereas there is no upper limit defining a minimum effectiveness. In the present dataset, no neural response could be detected in certain array contacts. In order to integrate these data points, the formula was adjusted so that zero defines minimum effectiveness. This allows the integration of contacts with no neural response, as they can be assigned a value of zero (minimal effectiveness). As the complete absence of a neural response for particular contacts is regularly observed in CI patients, we assume that this modification has the potential to render the here suggested efficiency index (EI) usable as an additional eCAP characteristic for clinical use.

In our experiments, the modified EI was significantly reduced in all regions of the basal modiolus, indicating a decrease in the efficiency of neural activation as a consequence of the laser-induced SGN ablation. This is in line with results from Konerding et al. (2025) [34], who employed the FI to demonstrate a reduced efficiency of neural activation after lesioning SGNs at the basal modiolus of guinea pigs.

### 4.5. Shifts in the eCAP Threshold Do Not Correlate with the Laser-Induced Damage Scores at the Modiolus

Consistent with Pfingst et al. (2015) [22] and Konerding et al. (2025) [34], our data do not suggest a correlation of the eCAP threshold with the weighted damage at the modiolus. This was expected, as the eCAP threshold reflects the contribution of a small, highly functional and synchronized subset of auditory nerve fibers rather than the state of the entire neural population. However, experiments in cats revealed that the threshold shifts and the range of affected frequencies increase in relation to the size of the lesion [29]. Nonetheless, in our experimental setting, the eCAP threshold was not a suitable characteristic to predict the preservation of SGNs. This could be caused by multiple factors influencing the eCAP threshold, including selective survival of functionally impaired neurons, the nonlinear summation properties of the threshold, or the electrode-neuron distance [46]. These factors could distort the relationship between the measured eCAP threshold and the actual number of functional SGNs, making the threshold an unsuitable eCAP characteristic to assess neural health in the cochlea.

### 4.6. ECAP Amplitude Shifts Correlate with Laser-Induced SGN Ablation in the Basal Modiolus

In our experiments, the observed changes in the eCAP amplitude were significantly correlated with the weighted SGN ablation at all regions of the modiolus [47]. This was expected, since the amplitude growth function, in contrast to the threshold, directly reflects the number of all synchronously firing neurons and thereby seems to be a suitable measure to assess neural health in the cochlea [20,48,49]. This is further supported by animal experiments showing a significantly higher amplitude in electrically evoked auditory brainstem response measurements in healthy compared to deafened cats, which were positively correlated with SGN density [50].

### 4.7. Shifts in the Efficiency Index Correlate with Damage Score in the Basal Modiolus

Consistent with the FI investigated by Konerding et al. (2025) [34], the EI showed a significant correlation with the extent of damage in all areas of the basal modiolus. Even though the capacity of the EI to predict the status of the SGNs is comparable to that of the amplitude, the EI appears to be superior, as the amplitude values are normalized by the applied current level. This renders the EI a controlled and thus more reliable parameter. We therefore conclude that the EI is currently the most accurate marker for evaluating neural health in the cochlea.

### 4.8. Potential Limitations and Clinical Translation

While the present animal experiments demonstrate that the eCAP amplitude and efficiency index are capable of assessing neural health under controlled experimental conditions, several limitations and challenges should be considered when translating this approach to cochlear implant (CI) patients. First, it remains unclear whether the laser-induced damage model applied in this study fully captures the inter-individual variability observed in CI patients with respect to etiology, duration of deafness, and central auditory plasticity. This variability may complicate both the interpretation and the clinical application of these measures in human subjects. Furthermore, as direct histological validation of neural health is not feasible in human CI users, clinical translation will require indirect validation using functional outcome measures such as speech perception, psychophysical thresholds, and imaging-based assessments of electrode position.

## 5. Conclusions

Taken together, our data suggest that the eCAP amplitude and the efficiency index could be useful characteristics for assessing neural health and identifying cochlear dead regions in the guinea pig. The present data suggest that modiolar dead regions could be detected by a local drop in eCAP amplitude values or the EI within the electrode array.

Future studies should evaluate the transferability of these objective intraoperative measurements for the identification of mDRs in CI patients. These findings, together with psychophysical measurements, may enable the identification and localization of mDRs, thus allowing for optimized CI fitting. Furthermore, animal experiments should be conducted in order to ascertain whether the implementation of these measurements could lead to a more precise determination of mDRs when a focused stimulation regimen is employed.

## Figures and Tables

**Table 1:** Classification and weighting system for laser-induced intracochlear damage at the basal modiolus.

Class	Characteristics	Weighting of Damage
1	Loss of <50% SGNs	length of damage × 1
2	Loss of ≥50% SGNs	length of damage × 2
3	Complete loss of SGNs or hearing nerve	length of damage × 3

## Data Availability

The raw data are available upon request due to legal (commercial) and ethical (animal research) reasons.

## Abbreviations

CG	Common ground
CI	Cochlear implant
CL	Current Level
E1–8	Electrode contact 1–8
eCAP	Electrically evoked compound action potential
MP1	Multi-polar 1
MP2	Multi-polar 2
mDR	Modiolar dead region
OC	Organ of Corti
RC	Rosenthal canal
RWM	Round window membrane
SGN	Spiral ganglion neuron
TIM	Transimpedance matrix
